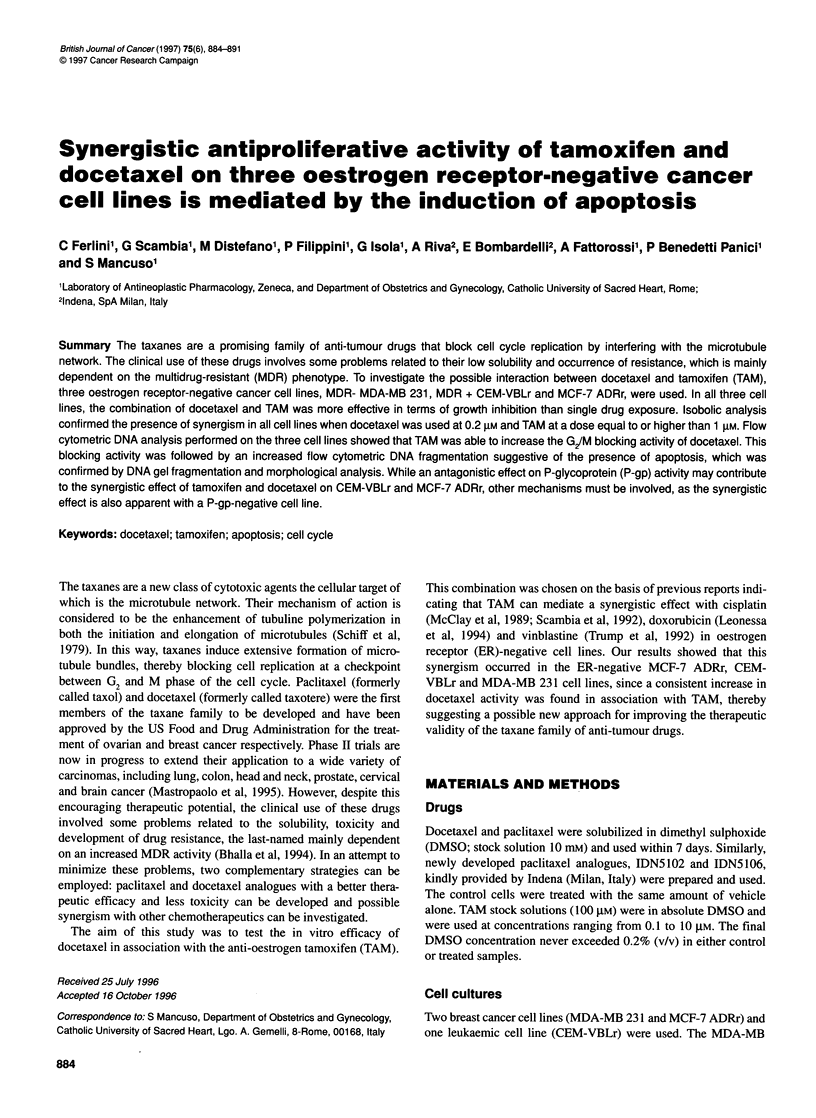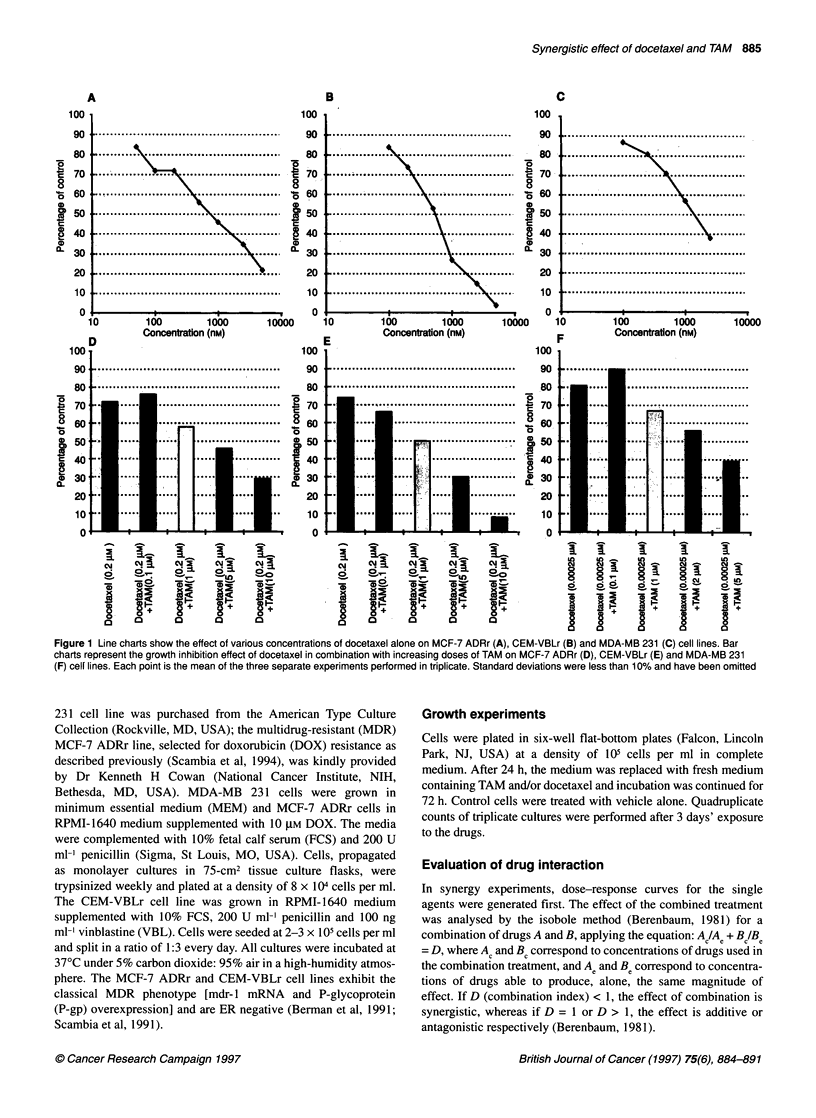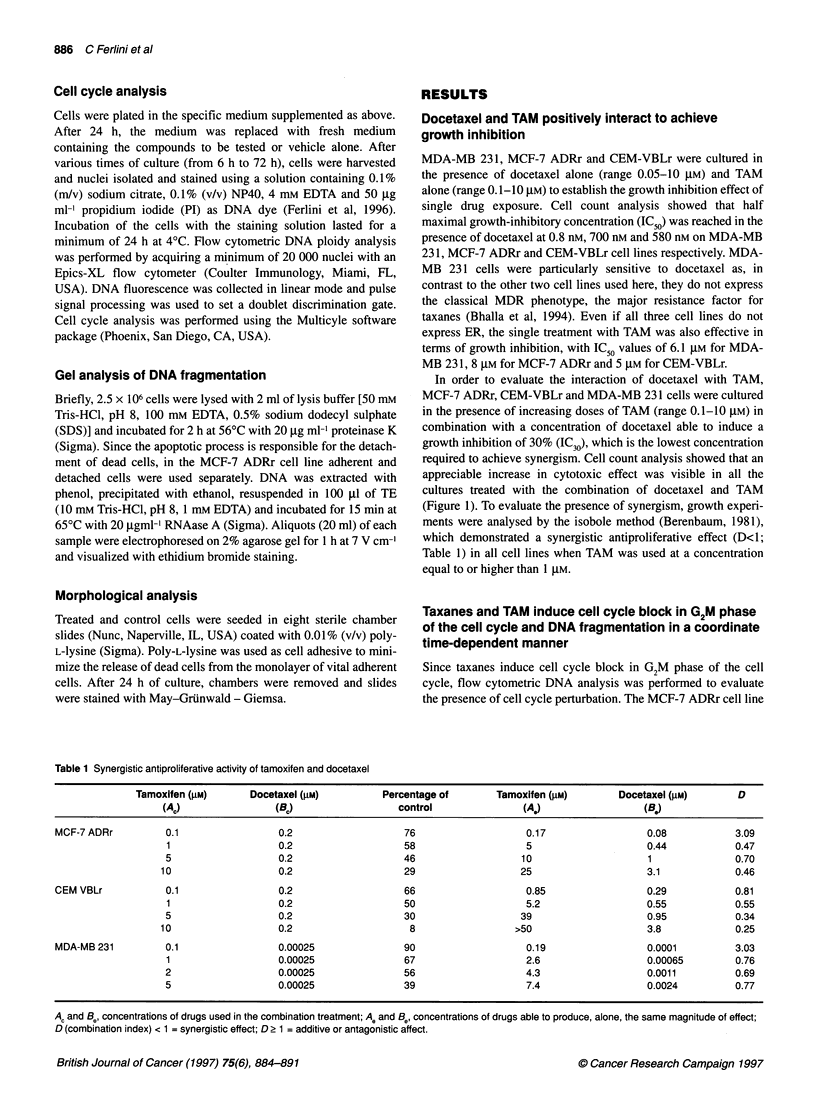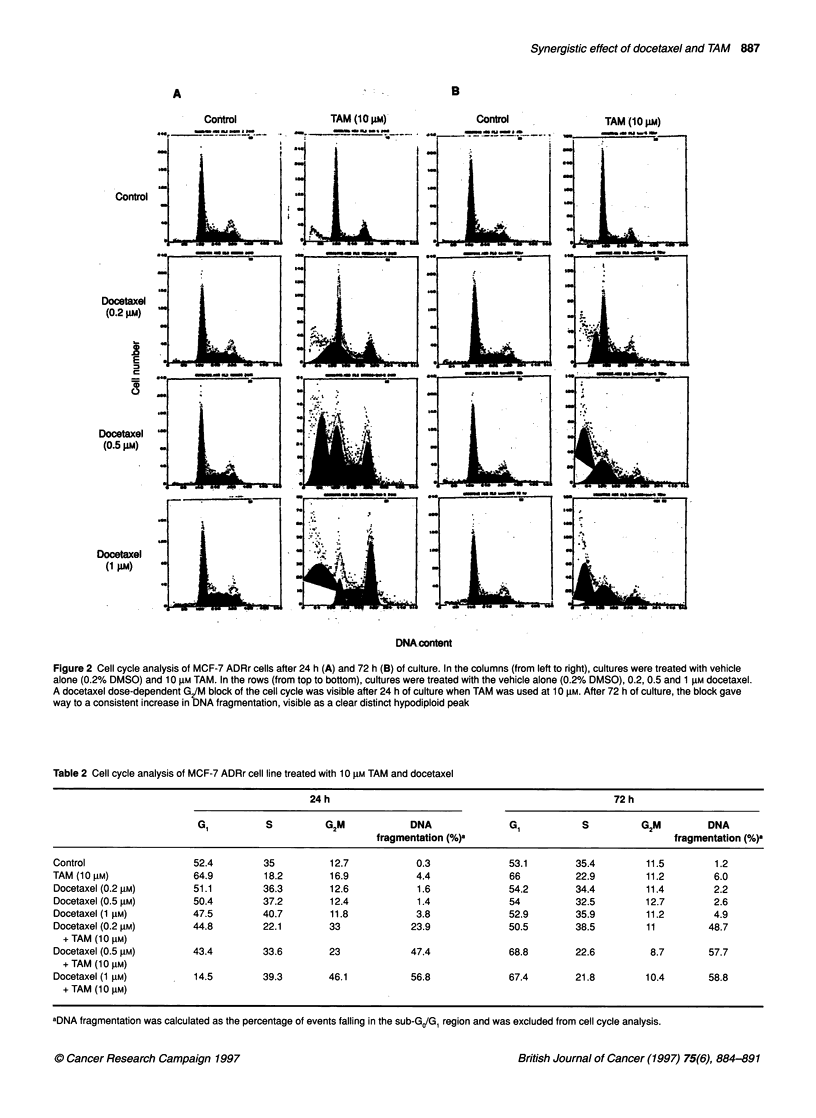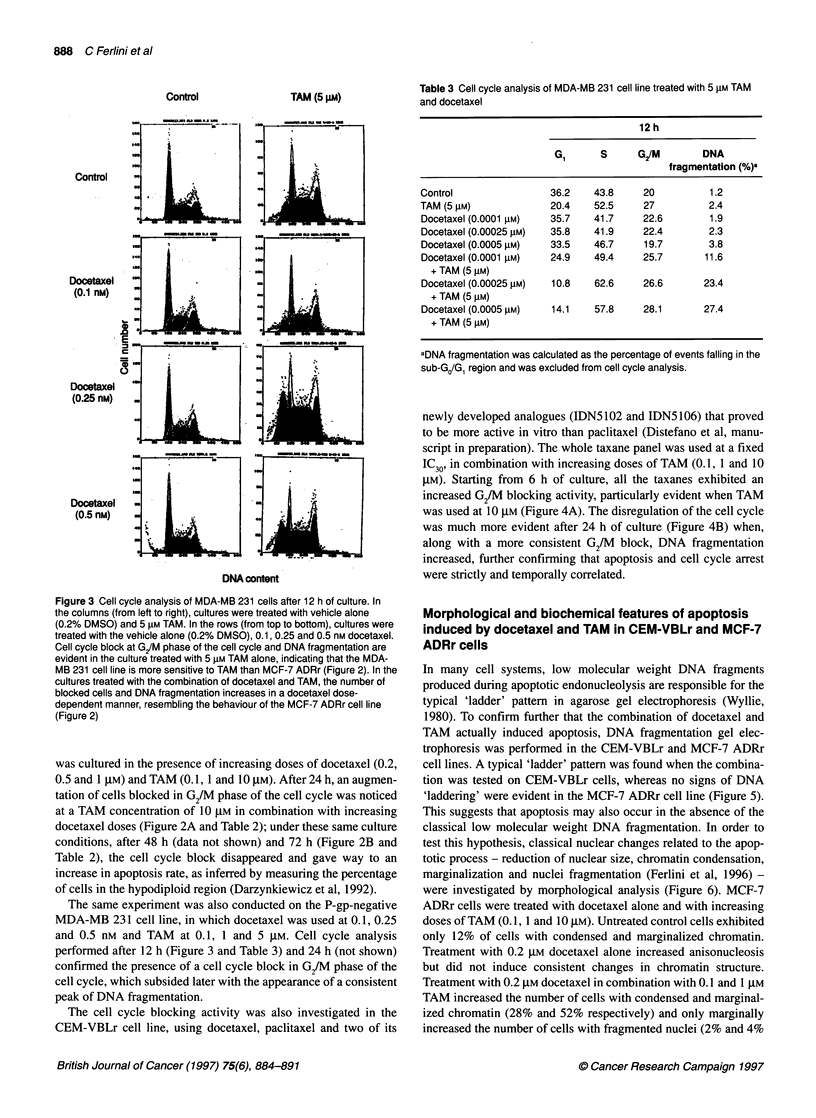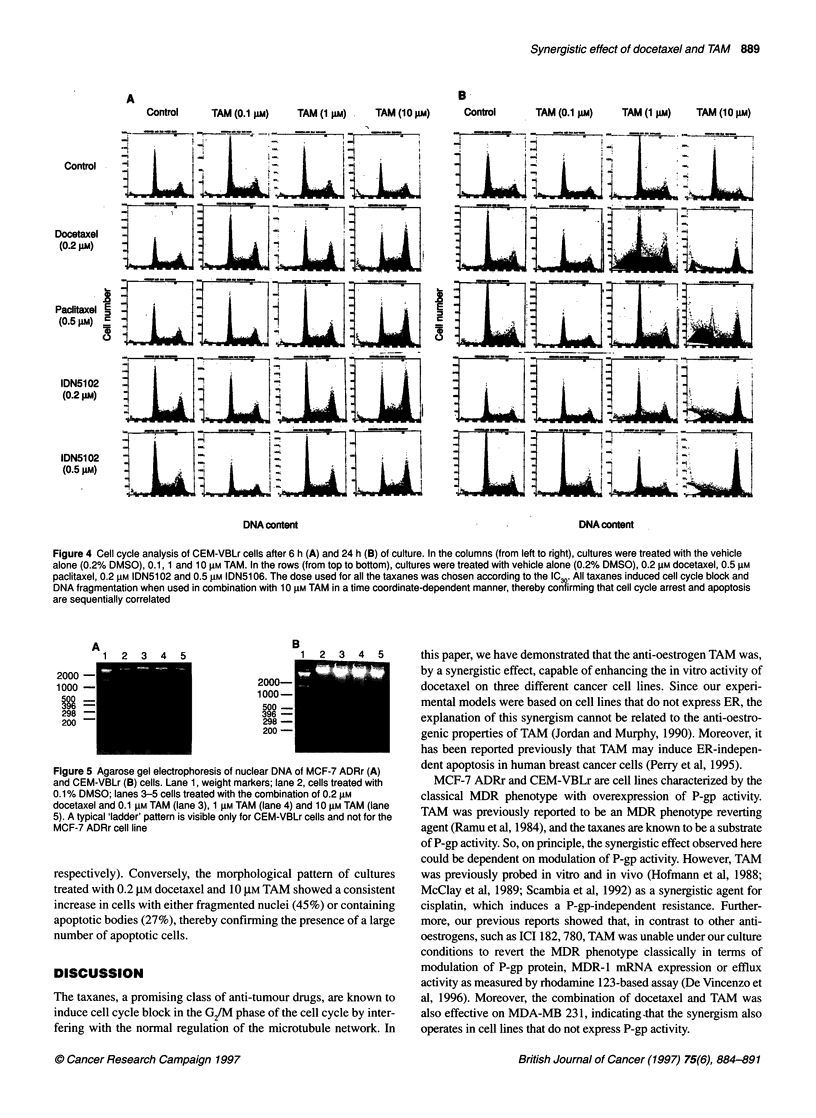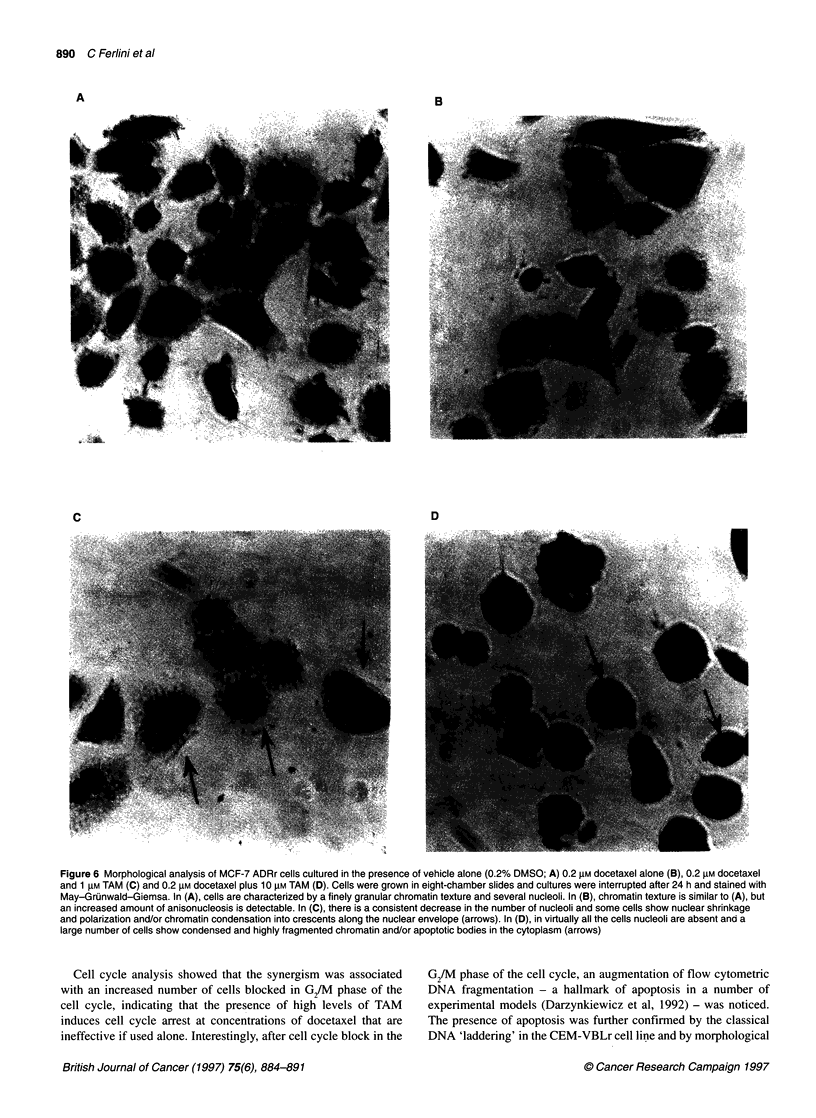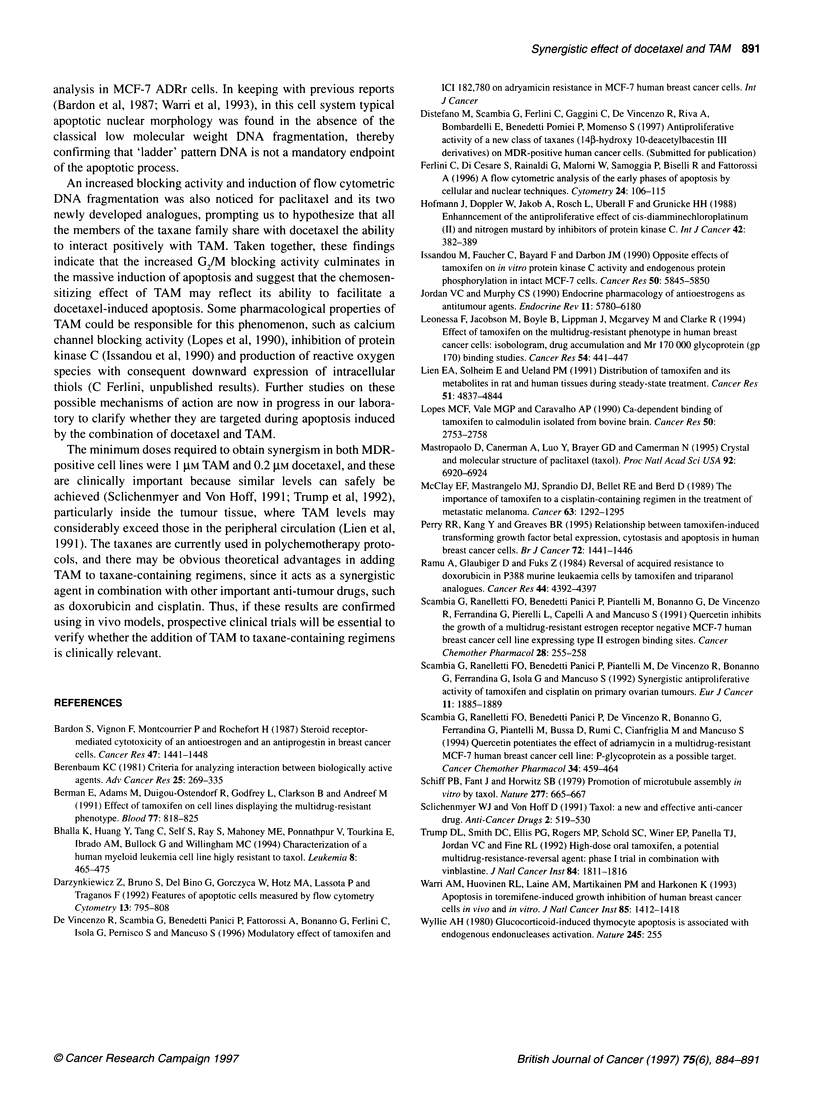# Synergistic antiproliferative activity of tamoxifen and docetaxel on three oestrogen receptor-negative cancer cell lines is mediated by the induction of apoptosis.

**DOI:** 10.1038/bjc.1997.156

**Published:** 1997

**Authors:** C. Ferlini, G. Scambia, M. Distefano, P. Filippini, G. Isola, A. Riva, E. Bombardelli, A. Fattorossi, P. Benedetti Panici, S. Mancuso

**Affiliations:** Department of Obstetrics and Gynecology, Catholic University of Sacred Heart, Rome, Italy.

## Abstract

**Images:**


British Journal of Cancer (1997) 75(6), 884–891
© 1997 Cancer Research Campaign

### Synergistic antiproliferative activity of tamoxifen and docetaxel on three oestrogen receptor-negative cancer cell lines is mediated by the induction of apoptosis

**C Ferlini[1], G Scambia[1], M Distefano[1], P Filippini[1], G Isola[1], A Riva[2], E Bombardelli[2], A Fattorossi[1], P Benedetti Panici[1] and S Mancuso[1]**

[1]Laboratory of Antineoplastic Pharmacology, Zeneca, and Department of Obstetrics and Gynecology, Catholic University of Sacred Heart, Rome; [2]Indena, SpA Milan, Italy

**Summary** The taxanes are a promising family of anti-tumour drugs that block cell cycle replication by interfering with the microtubule network. The clinical use of these drugs involves some problems related to their low solubility and occurrence of resistance, which is mainly dependent on the multidrug-resistant (MDR) phenotype. To investigate the possible interaction between docetaxel and tamoxifen (TAM), three oestrogen receptor-negative cancer cell lines, MDR- MDA-MB 231, MDR + CEM-VBLr and MCF-7 ADRr, were used. In all three cell lines, the combination of docetaxel and TAM was more effective in terms of growth inhibition than single drug exposure. Isobolic analysis confirmed the presence of synergism in all cell lines when docetaxel was used at 0.2 μM and TAM at a dose equal to or higher than 1 μM. Flow cytometric DNA analysis performed on the three cell lines showed that TAM was able to increase the $G_2$/M blocking activity of docetaxel. This blocking activity was followed by an increased flow cytometric DNA fragmentation suggestive of the presence of apoptosis, which was confirmed by DNA gel fragmentation and morphological analysis. While an antagonistic effect on P-glycoprotein (P-gp) activity may contribute to the synergistic effect of tamoxifen and docetaxel on CEM-VBLr and MCF-7 ADRr, other mechanisms must be involved, as the synergistic effect is also apparent with a P-gp-negative cell line.

**Keywords:** docetaxel; tamoxifen; apoptosis; cell cycle

The taxanes are a new class of cytotoxic agents the cellular target of which is the microtubule network. Their mechanism of action is considered to be the enhancement of tubuline polymerization in both the initiation and elongation of microtubules (Schiff et al, 1979). In this way, taxanes induce extensive formation of microtubule bundles, thereby blocking cell replication at a checkpoint between $G_2$ and M phase of the cell cycle. Paclitaxel (formerly called taxol) and docetaxel (formerly called taxotere) were the first members of the taxane family to be developed and have been approved by the US Food and Drug Administration for the treatment of ovarian and breast cancer respectively. Phase II trials are now in progress to extend their application to a wide variety of carcinomas, including lung, colon, head and neck, prostate, cervical and brain cancer (Mastropaolo et al, 1995). However, despite this encouraging therapeutic potential, the clinical use of these drugs involved some problems related to the solubility, toxicity and development of drug resistance, the last-named mainly dependent on an increased MDR activity (Bhalla et al, 1994). In an attempt to minimize these problems, two complementary strategies can be employed: paclitaxel and docetaxel analogues with a better therapeutic efficacy and less toxicity can be developed and possible synergism with other chemotherapeutics can be investigated.

The aim of this study was to test the in vitro efficacy of docetaxel in association with the anti-oestrogen tamoxifen (TAM). This combination was chosen on the basis of previous reports indicating that TAM can mediate a synergistic effect with cisplatin (McClay et al, 1989; Scambia et al, 1992), doxorubicin (Leonessa et al, 1994) and vinblastine (Trump et al, 1992) in oestrogen receptor (ER)-negative cell lines. Our results showed that this synergism occurred in the ER-negative MCF-7 ADRr, CEM-VBLr and MDA-MB 231 cell lines, since a consistent increase in docetaxel activity was found in association with TAM, thereby suggesting a possible new approach for improving the therapeutic validity of the taxane family of anti-tumour drugs.

*Received 25 July 1996*
*Accepted 16 October 1996*

*Correspondence to:* S Mancuso, Department of Obstetrics and Gynecology, Catholic University of Sacred Heart, Lgo. A. Gemelli, 8-Rome, 00168, Italy

### MATERIALS AND METHODS

### Drugs

Docetaxel and paclitaxel were solubilized in dimethyl sulphoxide (DMSO; stock solution 10 mM) and used within 7 days. Similarly, newly developed paclitaxel analogues, IDN5102 and IDN5106, kindly provided by Indena (Milan, Italy) were prepared and used. The control cells were treated with the same amount of vehicle alone. TAM stock solutions (100 μM) were in absolute DMSO and were used at concentrations ranging from 0.1 to 10 μM. The final DMSO concentration never exceeded 0.2% (v/v) in either control or treated samples.

### Cell cultures

Two breast cancer cell lines (MDA-MB 231 and MCF-7 ADRr) and one leukaemic cell line (CEM-VBLr) were used. The MDA-MB

**Figure 1** Line charts show the effect of various concentrations of docetaxel alone on MCF-7 ADRr (**A**), CEM-VBLr (**B**) and MDA-MB 231 (**C**) cell lines. Bar charts represent the growth inhibition effect of docetaxel in combination with increasing doses of TAM on MCF-7 ADRr (**D**), CEM-VBLr (**E**) and MDA-MB 231 (**F**) cell lines. Each point is the mean of the three separate experiments performed in triplicate. Standard deviations were less than 10% and have been omitted

231 cell line was purchased from the American Type Culture Collection (Rockville, MD, USA); the multidrug-resistant (MDR) MCF-7 ADRr line, selected for doxorubicin (DOX) resistance as described previously (Scambia et al, 1994), was kindly provided by Dr Kenneth H Cowan (National Cancer Institute, NIH, Bethesda, MD, USA). MDA-MB 231 cells were grown in minimum essential medium (MEM) and MCF-7 ADRr cells in RPMI-1640 medium supplemented with 10 µM DOX. The media were complemented with 10% fetal calf serum (FCS) and 200 U $ml^{-1}$ penicillin (Sigma, St Louis, MO, USA). Cells, propagated as monolayer cultures in 75-$cm^2$ tissue culture flasks, were trypsinized weekly and plated at a density of $8 \times 10^4$ cells per ml. The CEM-VBLr cell line was grown in RPMI-1640 medium supplemented with 10% FCS, 200 U $ml^{-1}$ penicillin and 100 ng $ml^{-1}$ vinblastine (VBL). Cells were seeded at $2–3 \times 10^5$ cells per ml and split in a ratio of 1:3 every day. All cultures were incubated at 37°C under 5% carbon dioxide: 95% air in a high-humidity atmosphere. The MCF-7 ADRr and CEM-VBLr cell lines exhibit the classical MDR phenotype [mdr-1 mRNA and P-glycoprotein (P-gp) overexpression] and are ER negative (Berman et al, 1991; Scambia et al, 1991).

### Growth experiments

Cells were plated in six-well flat-bottom plates (Falcon, Lincoln Park, NJ, USA) at a density of $10^5$ cells per ml in complete medium. After 24 h, the medium was replaced with fresh medium containing TAM and/or docetaxel and incubation was continued for 72 h. Control cells were treated with vehicle alone. Quadruplicate counts of triplicate cultures were performed after 3 days' exposure to the drugs.

### Evaluation of drug interaction

In synergy experiments, dose–response curves for the single agents were generated first. The effect of the combined treatment was analysed by the isobole method (Berenbaum, 1981) for a combination of drugs *A* and *B*, applying the equation: $A_c/A_e + B_c/B_e = D$, where $A_c$ and $B_c$ correspond to concentrations of drugs used in the combination treatment, and $A_e$ and $B_e$ correspond to concentrations of drugs able to produce, alone, the same magnitude of effect. If *D* (combination index) < 1, the effect of combination is synergistic, whereas if *D* = 1 or *D* > 1, the effect is additive or antagonistic respectively (Berenbaum, 1981).

© Cancer Research Campaign 1997

### Cell cycle analysis

Cells were plated in the specific medium supplemented as above. After 24 h, the medium was replaced with fresh medium containing the compounds to be tested or vehicle alone. After various times of culture (from 6 h to 72 h), cells were harvested and nuclei isolated and stained using a solution containing 0.1% (m/v) sodium citrate, 0.1% (v/v) NP40, 4 mM EDTA and 50 μg ml$^{-1}$ propidium iodide (PI) as DNA dye (Ferlini et al, 1996). Incubation of the cells with the staining solution lasted for a minimum of 24 h at 4°C. Flow cytometric DNA ploidy analysis was performed by acquiring a minimum of 20 000 nuclei with an Epics-XL flow cytometer (Coulter Immunology, Miami, FL, USA). DNA fluorescence was collected in linear mode and pulse signal processing was used to set a doublet discrimination gate. Cell cycle analysis was performed using the Multicyle software package (Phoenix, San Diego, CA, USA).

### Gel analysis of DNA fragmentation

Briefly, 2.5 × 10$^6$ cells were lysed with 2 ml of lysis buffer [50 mM Tris-HCl, pH 8, 100 mM EDTA, 0.5% sodium dodecyl sulphate (SDS)] and incubated for 2 h at 56°C with 20 μg ml$^{-1}$ proteinase K (Sigma). Since the apoptotic process is responsible for the detachment of dead cells, in the MCF-7 ADRr cell line adherent and detached cells were used separately. DNA was extracted with phenol, precipitated with ethanol, resuspended in 100 μl of TE (10 mM Tris-HCl, pH 8, 1 mM EDTA) and incubated for 15 min at 65°C with 20 μgml$^{-1}$ RNAase A (Sigma). Aliquots (20 ml) of each sample were electrophoresed on 2% agarose gel for 1 h at 7 V cm$^{-1}$ and visualized with ethidium bromide staining.

### Morphological analysis

Treated and control cells were seeded in eight sterile chamber slides (Nunc, Naperville, IL, USA) coated with 0.01% (v/v) poly-L-lysine (Sigma). Poly-L-lysine was used as cell adhesive to minimize the release of dead cells from the monolayer of vital adherent cells. After 24 h of culture, chambers were removed and slides were stained with May–Grünwald – Giemsa.

### RESULTS

### Docetaxel and TAM positively interact to achieve growth inhibition

MDA-MB 231, MCF-7 ADRr and CEM-VBLr were cultured in the presence of docetaxel alone (range 0.05–10 μM) and TAM alone (range 0.1–10 μM) to establish the growth inhibition effect of single drug exposure. Cell count analysis showed that half maximal growth-inhibitory concentration ($IC_{50}$) was reached in the presence of docetaxel at 0.8 nM, 700 nM and 580 nM on MDA-MB 231, MCF-7 ADRr and CEM-VBLr cell lines respectively. MDA-MB 231 cells were particularly sensitive to docetaxel as, in contrast to the other two cell lines used here, they do not express the classical MDR phenotype, the major resistance factor for taxanes (Bhalla et al, 1994). Even if all three cell lines do not express ER, the single treatment with TAM was also effective in terms of growth inhibition, with $IC_{50}$ values of 6.1 μM for MDA-MB 231, 8 μM for MCF-7 ADRr and 5 μM for CEM-VBLr.

In order to evaluate the interaction of docetaxel with TAM, MCF-7 ADRr, CEM-VBLr and MDA-MB 231 cells were cultured in the presence of increasing doses of TAM (range 0.1–10 μM) in combination with a concentration of docetaxel able to induce a growth inhibition of 30% ($IC_{30}$), which is the lowest concentration required to achieve synergism. Cell count analysis showed that an appreciable increase in cytotoxic effect was visible in all the cultures treated with the combination of docetaxel and TAM (Figure 1). To evaluate the presence of synergism, growth experiments were analysed by the isobole method (Berenbaum, 1981), which demonstrated a synergistic antiproliferative effect (D<1; Table 1) in all cell lines when TAM was used at a concentration equal to or higher than 1 μM.

### Taxanes and TAM induce cell cycle block in $G_2M$ phase of the cell cycle and DNA fragmentation in a coordinate time-dependent manner

Since taxanes induce cell cycle block in $G_2M$ phase of the cell cycle, flow cytometric DNA analysis was performed to evaluate the presence of cell cycle perturbation. The MCF-7 ADRr cell line

**Table 1** Synergistic antiproliferative activity of tamoxifen and docetaxel

| | Tamoxifen (μM) ($A_c$) | Docetaxel (μM) ($B_c$) | Percentage of control | Tamoxifen (μM) ($A_e$) | Docetaxel (μM) ($B_e$) | *D* |
|---|---|---|---|---|---|---|
| MCF-7 ADRr | 0.1 | 0.2 | 76 | 0.17 | 0.08 | 3.09 |
| | 1 | 0.2 | 58 | 5 | 0.44 | 0.47 |
| | 5 | 0.2 | 46 | 10 | 1 | 0.70 |
| | 10 | 0.2 | 29 | 25 | 3.1 | 0.46 |
| CEM VBLr | 0.1 | 0.2 | 66 | 0.85 | 0.29 | 0.81 |
| | 1 | 0.2 | 50 | 5.2 | 0.55 | 0.55 |
| | 5 | 0.2 | 30 | 39 | 0.95 | 0.34 |
| | 10 | 0.2 | 8 | >50 | 3.8 | 0.25 |
| MDA-MB 231 | 0.1 | 0.00025 | 90 | 0.19 | 0.0001 | 3.03 |
| | 1 | 0.00025 | 67 | 2.6 | 0.00065 | 0.76 |
| | 2 | 0.00025 | 56 | 4.3 | 0.0011 | 0.69 |
| | 5 | 0.00025 | 39 | 7.4 | 0.0024 | 0.77 |

$A_c$ and $B_e$, concentrations of drugs used in the combination treatment; $A_e$ and $B_e$, concentrations of drugs able to produce, alone, the same magnitude of effect; *D* (combination index) < 1 = synergistic effect; $D \geq 1$ = additive or antagonistic affect.

© Cancer Research Campaign 1997

**Figure 2** Cell cycle analysis of MCF-7 ADRr cells after 24 h (**A**) and 72 h (**B**) of culture. In the columns (from left to right), cultures were treated with vehicle alone (0.2% DMSO) and 10 µM TAM. In the rows (from top to bottom), cultures were treated with the vehicle alone (0.2% DMSO), 0.2, 0.5 and 1 µM docetaxel. A docetaxel dose-dependent $G_2$/M block of the cell cycle was visible after 24 h of culture when TAM was used at 10 µM. After 72 h of culture, the block gave way to a consistent increase in DNA fragmentation, visible as a clear distinct hypodiploid peak

**Table 2** Cell cycle analysis of MCF-7 ADRr cell line treated with 10 µM TAM and docetaxel

| | 24 h | | | | 72 h | | | |
|---|---|---|---|---|---|---|---|---|
| | $G_1$ | S | $G_2$M | DNA fragmentation (%)[a] | $G_1$ | S | $G_2$M | DNA fragmentation (%)[a] |
| Control | 52.4 | 35 | 12.7 | 0.3 | 53.1 | 35.4 | 11.5 | 1.2 |
| TAM (10 µM) | 64.9 | 18.2 | 16.9 | 4.4 | 66 | 22.9 | 11.2 | 6.0 |
| Docetaxel (0.2 µM) | 51.1 | 36.3 | 12.6 | 1.6 | 54.2 | 34.4 | 11.4 | 2.2 |
| Docetaxel (0.5 µM) | 50.4 | 37.2 | 12.4 | 1.4 | 54 | 32.5 | 12.7 | 2.6 |
| Docetaxel (1 µM) | 47.5 | 40.7 | 11.8 | 3.8 | 52.9 | 35.9 | 11.2 | 4.9 |
| Docetaxel (0.2 µM) + TAM (10 µM) | 44.8 | 22.1 | 33 | 23.9 | 50.5 | 38.5 | 11 | 48.7 |
| Docetaxel (0.5 µM) + TAM (10 µM) | 43.4 | 33.6 | 23 | 47.4 | 68.8 | 22.6 | 8.7 | 57.7 |
| Docetaxel (1 µM) + TAM (10 µM) | 14.5 | 39.3 | 46.1 | 56.8 | 67.4 | 21.8 | 10.4 | 58.8 |

[a]DNA fragmentation was calculated as the percentage of events falling in the sub-$G_0$/$G_1$ region and was excluded from cell cycle analysis.

**Figure 3** Cell cycle analysis of MDA-MB 231 cells after 12 h of culture. In the columns (from left to right), cultures were treated with vehicle alone (0.2% DMSO) and 5 μM TAM. In the rows (from top to bottom), cultures were treated with the vehicle alone (0.2% DMSO), 0.1, 0.25 and 0.5 nM docetaxel. Cell cycle block at $G_2$/M phase of the cell cycle and DNA fragmentation are evident in the culture treated with 5 μM TAM alone, indicating that the MDA-MB 231 cell line is more sensitive to TAM than MCF-7 ADRr (Figure 2). In the cultures treated with the combination of docetaxel and TAM, the number of blocked cells and DNA fragmentation increases in a docetaxel dose-dependent manner, resembling the behaviour of the MCF-7 ADRr cell line (Figure 2)

was cultured in the presence of increasing doses of docetaxel (0.2, 0.5 and 1 μM) and TAM (0.1, 1 and 10 μM). After 24 h, an augmentation of cells blocked in $G_2$/M phase of the cell cycle was noticed at a TAM concentration of 10 μM in combination with increasing docetaxel doses (Figure 2A and Table 2); under these same culture conditions, after 48 h (data not shown) and 72 h (Figure 2B and Table 2), the cell cycle block disappeared and gave way to an increase in apoptosis rate, as inferred by measuring the percentage of cells in the hypodiploid region (Darzynkiewicz et al, 1992).

The same experiment was also conducted on the P-gp-negative MDA-MB 231 cell line, in which docetaxel was used at 0.1, 0.25 and 0.5 nM and TAM at 0.1, 1 and 5 μM. Cell cycle analysis performed after 12 h (Figure 3 and Table 3) and 24 h (not shown) confirmed the presence of a cell cycle block in $G_2$/M phase of the cell cycle, which subsided later with the appearance of a consistent peak of DNA fragmentation.

The cell cycle blocking activity was also investigated in the CEM-VBLr cell line, using docetaxel, paclitaxel and two of its

**Table 3** Cell cycle analysis of MDA-MB 231 cell line treated with 5 μM TAM and docetaxel

| | 12 h | | | |
|---|---|---|---|---|
| | $G_1$ | S | $G_2$/M | DNA fragmentation (%)[a] |
| Control | 36.2 | 43.8 | 20 | 1.2 |
| TAM (5 μM) | 20.4 | 52.5 | 27 | 2.4 |
| Docetaxel (0.0001 μM) | 35.7 | 41.7 | 22.6 | 1.9 |
| Docetaxel (0.00025 μM) | 35.8 | 41.9 | 22.4 | 2.3 |
| Docetaxel (0.0005 μM) | 33.5 | 46.7 | 19.7 | 3.8 |
| Docetaxel (0.0001 μM) + TAM (5 μM) | 24.9 | 49.4 | 25.7 | 11.6 |
| Docetaxel (0.00025 μM) + TAM (5 μM) | 10.8 | 62.6 | 26.6 | 23.4 |
| Docetaxel (0.0005 μM) + TAM (5 μM) | 14.1 | 57.8 | 28.1 | 27.4 |

[a]DNA fragmentation was calculated as the percentage of events falling in the sub-$G_0$/$G_1$ region and was excluded from cell cycle analysis.

newly developed analogues (IDN5102 and IDN5106) that proved to be more active in vitro than paclitaxel (Distefano et al, manuscript in preparation). The whole taxane panel was used at a fixed $IC_{30}$, in combination with increasing doses of TAM (0.1, 1 and 10 μM). Starting from 6 h of culture, all the taxanes exhibited an increased $G_2$/M blocking activity, particularly evident when TAM was used at 10 μM (Figure 4A). The disregulation of the cell cycle was much more evident after 24 h of culture (Figure 4B) when, along with a more consistent $G_2$/M block, DNA fragmentation increased, further confirming that apoptosis and cell cycle arrest were strictly and temporally correlated.

### Morphological and biochemical features of apoptosis induced by docetaxel and TAM in CEM-VBLr and MCF-7 ADRr cells

In many cell systems, low molecular weight DNA fragments produced during apoptotic endonucleolysis are responsible for the typical 'ladder' pattern in agarose gel electrophoresis (Wyllie, 1980). To confirm further that the combination of docetaxel and TAM actually induced apoptosis, DNA fragmentation gel electrophoresis was performed in the CEM-VBLr and MCF-7 ADRr cell lines. A typical 'ladder' pattern was found when the combination was tested on CEM-VBLr cells, whereas no signs of DNA 'laddering' were evident in the MCF-7 ADRr cell line (Figure 5). This suggests that apoptosis may also occur in the absence of the classical low molecular weight DNA fragmentation. In order to test this hypothesis, classical nuclear changes related to the apoptotic process – reduction of nuclear size, chromatin condensation, marginalization and nuclei fragmentation (Ferlini et al, 1996) – were investigated by morphological analysis (Figure 6). MCF-7 ADRr cells were treated with docetaxel alone and with increasing doses of TAM (0.1, 1 and 10 μM). Untreated control cells exhibited only 12% of cells with condensed and marginalized chromatin. Treatment with 0.2 μM docetaxel alone increased anisonucleosis but did not induce consistent changes in chromatin structure. Treatment with 0.2 μM docetaxel in combination with 0.1 and 1 μM TAM increased the number of cells with condensed and marginalized chromatin (28% and 52% respectively) and only marginally increased the number of cells with fragmented nuclei (2% and 4%

© Cancer Research Campaign 1997

**Figure 4** Cell cycle analysis of CEM-VBLr cells after 6 h (**A**) and 24 h (**B**) of culture. In the columns (from left to right), cultures were treated with the vehicle alone (0.2% DMSO), 0.1, 1 and 10 μM TAM. In the rows (from top to bottom), cultures were treated with vehicle alone (0.2% DMSO), 0.2 μM docetaxel, 0.5 μM paclitaxel, 0.2 μM IDN5102 and 0.5 μM IDN5106. The dose used for all the taxanes was chosen according to the $IC_{30}$. All taxanes induced cell cycle block and DNA fragmentation when used in combination with 10 μM TAM in a time coordinate-dependent manner, thereby confirming that cell cycle arrest and apoptosis are sequentially correlated

**Figure 5** Agarose gel electrophoresis of nuclear DNA of MCF-7 ADRr (**A**) and CEM-VBLr (**B**) cells. Lane 1, weight markers; lane 2, cells treated with 0.1% DMSO; lanes 3–5 cells treated with the combination of 0.2 μM docetaxel and 0.1 μM TAM (lane 3), 1 μM TAM (lane 4) and 10 μM TAM (lane 5). A typical 'ladder' pattern is visible only for CEM-VBLr cells and not for the MCF-7 ADRr cell line respectively). Conversely, the morphological pattern of cultures treated with 0.2 μM docetaxel and 10 μM TAM showed a consistent increase in cells with either fragmented nuclei (45%) or containing apoptotic bodies (27%), thereby confirming the presence of a large number of apoptotic cells.

### DISCUSSION

The taxanes, a promising class of anti-tumour drugs, are known to induce cell cycle block in the $G_2$/M phase of the cell cycle by interfering with the normal regulation of the microtubule network. In this paper, we have demonstrated that the anti-oestrogen TAM was, by a synergistic effect, capable of enhancing the in vitro activity of docetaxel on three different cancer cell lines. Since our experimental models were based on cell lines that do not express ER, the explanation of this synergism cannot be related to the anti-oestrogenic properties of TAM (Jordan and Murphy, 1990). Moreover, it has been reported previously that TAM may induce ER-independent apoptosis in human breast cancer cells (Perry et al, 1995).

MCF-7 ADRr and CEM-VBLr are cell lines characterized by the classical MDR phenotype with overexpression of P-gp activity. TAM was previously reported to be an MDR phenotype reverting agent (Ramu et al, 1984), and the taxanes are known to be a substrate of P-gp activity. So, on principle, the synergistic effect observed here could be dependent on modulation of P-gp activity. However, TAM was previously probed in vitro and in vivo (Hofmann et al, 1988; McClay et al, 1989; Scambia et al, 1992) as a synergistic agent for cisplatin, which induces a P-gp-independent resistance. Furthermore, our previous reports showed that, in contrast to other anti-oestrogens, such as ICI 182, 780, TAM was unable under our culture conditions to revert the MDR phenotype classically in terms of modulation of P-gp protein, MDR-1 mRNA expression or efflux activity as measured by rhodamine 123-based assay (De Vincenzo et al, 1996). Moreover, the combination of docetaxel and TAM was also effective on MDA-MB 231, indicating that the synergism also operates in cell lines that do not express P-gp activity.

© Cancer Research Campaign 1997

**Figure 6** Morphological analysis of MCF-7 ADRr cells cultured in the presence of vehicle alone (0.2% DMSO; **A**) 0.2 μM docetaxel alone (**B**), 0.2 μM docetaxel and 1 μM TAM (**C**) and 0.2 μM docetaxel plus 10 μM TAM (**D**). Cells were grown in eight-chamber slides and cultures were interrupted after 24 h and stained with May–Grünwald–Giemsa. In (**A**), cells are characterized by a finely granular chromatin texture and several nucleoli. In (**B**), chromatin texture is similar to (**A**), but an increased amount of anisonucleosis is detectable. In (**C**), there is a consistent decrease in the number of nucleoli and some cells show nuclear shrinkage and polarization and/or chromatin condensation into crescents along the nuclear envelope (arrows). In (**D**), in virtually all the cells nucleoli are absent and a large number of cells show condensed and highly fragmented chromatin and/or apoptotic bodies in the cytoplasm (arrows)

Cell cycle analysis showed that the synergism was associated with an increased number of cells blocked in $G_2$/M phase of the cell cycle, indicating that the presence of high levels of TAM induces cell cycle arrest at concentrations of docetaxel that are ineffective if used alone. Interestingly, after cell cycle block in the $G_2$/M phase of the cell cycle, an augmentation of flow cytometric DNA fragmentation – a hallmark of apoptosis in a number of experimental models (Darzynkiewicz et al, 1992) – was noticed. The presence of apoptosis was further confirmed by the classical DNA 'laddering' in the CEM-VBLr cell line and by morphological

© Cancer Research Campaign 1997

analysis in MCF-7 ADRr cells. In keeping with previous reports (Bardon et al, 1987; Warri et al, 1993), in this cell system typical apoptotic nuclear morphology was found in the absence of the classical low molecular weight DNA fragmentation, thereby confirming that 'ladder' pattern DNA is not a mandatory endpoint of the apoptotic process.

An increased blocking activity and induction of flow cytometric DNA fragmentation was also noticed for paclitaxel and its two newly developed analogues, prompting us to hypothesize that all the members of the taxane family share with docetaxel the ability to interact positively with TAM. Taken together, these findings indicate that the increased $G_2/M$ blocking activity culminates in the massive induction of apoptosis and suggest that the chemosensitizing effect of TAM may reflect its ability to facilitate a docetaxel-induced apoptosis. Some pharmacological properties of TAM could be responsible for this phenomenon, such as calcium channel blocking activity (Lopes et al, 1990), inhibition of protein kinase C (Issandou et al, 1990) and production of reactive oxygen species with consequent downward expression of intracellular thiols (C Ferlini, unpublished results). Further studies on these possible mechanisms of action are now in progress in our laboratory to clarify whether they are targeted during apoptosis induced by the combination of docetaxel and TAM.

The minimum doses required to obtain synergism in both MDR-positive cell lines were 1 μM TAM and 0.2 μM docetaxel, and these are clinically important because similar levels can safely be achieved (Sclichenmyer and Von Hoff, 1991; Trump et al, 1992), particularly inside the tumour tissue, where TAM levels may considerably exceed those in the peripheral circulation (Lien et al, 1991). The taxanes are currently used in polychemotherapy protocols, and there may be obvious theoretical advantages in adding TAM to taxane-containing regimens, since it acts as a synergistic agent in combination with other important anti-tumour drugs, such as doxorubicin and cisplatin. Thus, if these results are confirmed using in vivo models, prospective clinical trials will be essential to verify whether the addition of TAM to taxane-containing regimens is clinically relevant.

### REFERENCES

Bardon S, Vignon F, Montcourrier P and Rochefort H (1987) Steroid receptor-mediated cytotoxicity of an antioestrogen and an antiprogestin in breast cancer cells. *Cancer Res* **47**: 1441–1448

Berenbaum KC (1981) Criteria for analyzing interaction between biologically active agents. *Adv Cancer Res* **25**: 269–335

Berman E, Adams M, Duigou-Ostendorf R, Godfrey L, Clarkson B and Andreef M (1991) Effect of tamoxifen on cell lines displaying the multidrug-resistant phenotype. *Blood* **77**: 818–825

Bhalla K, Huang Y, Tang C, Self S, Ray S, Mahoney ME, Ponnathpur V, Tourkina E, Ibrado AM, Bullock G and Willingham MC (1994) Characterization of a human myeloid leukemia cell line higly resistant to taxol. *Leukemia* **8**: 465–475

Darzynkiewicz Z, Bruno S, Del Bino G, Gorczyca W, Hotz MA, Lassota P and Traganos F (1992) Features of apoptotic cells measured by flow cytometry *Cytometry* **13**: 795–808

De Vincenzo R, Scambia G, Benedetti Panici P, Fattorossi A, Bonanno G, Ferlini C, Isola G, Pernisco S and Mancuso S (1996) Modulatory effect of tamoxifen and ICI 182,780 on adryamicin resistance in MCF-7 human breast cancer cells. *Int J Cancer*

Distefano M, Scambia G, Ferlini C, Gaggini C, De Vincenzo R, Riva A, Bombardelli E, Benedetti Pomiei P, Momenso S (1997) Antiproliferative activity of a new class of taxanes (14β-hydroxy 10-deacetylbacestin III derivatives) on MDR-positive human cancer cells. (Submitted for publication)

Ferlini C, Di Cesare S, Rainaldi G, Malorni W, Samoggia P, Biselli R and Fattorossi A (1996) A flow cytometric analysis of the early phases of apoptosis by cellular and nuclear techniques. *Cytometry* **24**: 106–115

Hofmann J, Doppler W, Jakob A, Rosch L, Uberall F and Grunicke HH (1988) Enhanncement of the antiproliferative effect of cis-diamminechloroplatinum (II) and nitrogen mustard by inhibitors of protein kinase C. *Int J Cancer* **42**: 382–389

Issandou M, Faucher C, Bayard F and Darbon JM (1990) Opposite effects of tamoxifen on *in vitro* protein kinase C activity and endogenous protein phosphorylation in intact MCF-7 cells. *Cancer Res* **50**: 5845–5850

Jordan VC and Murphy CS (1990) Endocrine pharmacology of antioestrogens as antitumour agents. *Endocrine Rev* **11**: 5780–6180

Leonessa F, Jacobson M, Boyle B, Lippman J, Mcgarvey M and Clarke R (1994) Effect of tamoxifen on the multidrug-resistant phenotype in human breast cancer cells: isobologram, drug accumulation and Mr 170 000 glycoprotein (gp 170) binding studies. *Cancer Res* **54**: 441–447

Lien EA, Solheim E and Ueland PM (1991) Distribution of tamoxifen and its metabolites in rat and human tissues during steady-state treatment. *Cancer Res* **51**: 4837–4844

Lopes MCF, Vale MGP and Caravalho AP (1990) Ca-dependent binding of tamoxifen to calmodulin isolated from bovine brain. *Cancer Res* **50**: 2753–2758

Mastropaolo D, Canerman A, Luo Y, Brayer GD and Camerman N (1995) Crystal and molecular structure of paclitaxel (taxol). *Proc Natl Acad Sci USA* **92**: 6920–6924

McClay EF, Mastrangelo MJ, Sprandio DJ, Bellet RE and Berd D (1989) The importance of tamoxifen to a cisplatin-containing regimen in the treatment of metastatic melanoma. *Cancer* **63**: 1292–1295

Perry RR, Kang Y and Greaves BR (1995) Relationship between tamoxifen-induced transforming growth factor betal expression, cytostasis and apoptosis in human breast cancer cells. *Br J Cancer* **72**: 1441–1446

Ramu A, Glaubiger D and Fuks Z (1984) Reversal of acquired resistance to doxorubicin in P388 murine leukaemia cells by tamoxifen and triparanol analogues. *Cancer Res* **44**: 4392–4397

Scambia G, Ranelletti FO, Benedetti Panici P, Piantelli M, Bonanno G, De Vincenzo R, Ferrandina G, Pierelli L, Capelli A and Mancuso S (1991) Quercetin inhibits the growth of a multidrug-resistant estrogen receptor negative MCF-7 human breast cancer cell line expressing type II estrogen binding sites. *Cancer Chemother Pharmacol* **28**: 255–258

Scambia G, Ranelletti FO, Benedetti Panici P, Piantelli M, De Vincenzo R, Bonanno G, Ferrandina G, Isola G and Mancuso S (1992) Synergistic antiproliferative activity of tamoxifen and cisplatin on primary ovarian tumours. *Eur J Cancer* **11**: 1885–1889

Scambia G, Ranelletti FO, Benedetti Panici P, De Vincenzo R, Bonanno G, Ferrandina G, Piantelli M, Bussa D, Rumi C, Cianfriglia M and Mancuso S (1994) Quercetin potentiates the effect of adriamycin in a multidrug-resistant MCF-7 human breast cancer cell line: P-glycoprotein as a possible target. *Cancer Chemother Pharmacol* **34**: 459–464

Schiff PB, Fant J and Horwitz SB (1979) Promotion of microtubule assembly *in vitro* by taxol. *Nature* **277**: 665–667

Sclichenmyer WJ and Von Hoff D (1991) Taxol: a new and effective anti-cancer drug. *Anti-Cancer Drugs* **2**: 519–530

Trump DL, Smith DC, Ellis PG, Rogers MP, Schold SC, Winer EP, Panella TJ, Jordan VC and Fine RL (1992) High-dose oral tamoxifen, a potential multidrug-resistance-reversal agent: phase I trial in combination with vinblastine. *J Natl Cancer Inst* **84**: 1811–1816

Warri AM, Huovinen RL, Laine AM, Martikainen PM and Harkonen K (1993) Apoptosis in toremifene-induced growth inhibition of human breast cancer cells *in vivo* and *in vitro*. *J Natl Cancer Inst* **85**: 1412–1418

Wyllie AH (1980) Glucocorticoid-induced thymocyte apoptosis is associated with endogenous endonucleases activation. *Nature* **245**: 255

© Cancer Research Campaign 1997